# Acute Reactogenicity after Intramuscular Immunization with Recombinant Vesicular Stomatitis Virus Is Linked to Production of IL-1β

**DOI:** 10.1371/journal.pone.0046516

**Published:** 2012-10-08

**Authors:** Kathleen Athearn, Christopher J. Sample, Brice E. Barefoot, Kristi L. Williams, Elizabeth A. Ramsburg

**Affiliations:** 1 Human Vaccine Institute, School of Medicine, Duke University, Durham, North Carolina, United States of America; 2 Department of Medicine, School of Medicine, Duke University, Durham, North Carolina, United States of America; 3 Department of Pathology, School of Medicine, Duke University, Durham, North Carolina, United States of America; 4 School of Nursing, School of Medicine, Duke University, Durham, North Carolina, United States of America; Instituto Butantan, Brazil

## Abstract

Vaccines based on live viruses are attractive because they are immunogenic, cost-effective, and can be delivered by multiple routes. However, live virus vaccines also cause reactogenic side effects such as fever, myalgia, and injection site pain that have reduced their acceptance in the clinic. Several recent studies have linked vaccine-induced reactogenic side effects to production of the pro-inflammatory cytokine interleukin-1β (IL-1β) in humans. Our objective was therefore to determine whether IL-1β contributed to pathology after immunization with recombinant vesicular stomatitis virus (rVSV) vaccine vectors, and if so, to identify strategies by which IL-1β mediated pathology might be reduced without compromising immunogenicity. We found that an rVSV vaccine induced local and systemic production of IL-1β *in vivo*, and that accumulation of IL-1β correlated with acute pathology after rVSV immunization. rVSV-induced pathology was reduced in mice deficient in the IL-1 receptor Type I, but the IL-1R−/− mice were fully protected from lethal rechallenge with a high dose of VSV. This result demonstrated that IL-1 contributed to reactogenicity of the rVSV, but was dispensable for induction of protective immunity. The amount of IL-1β detected in mice deficient in either caspase-1 or the inflammasome adaptor molecule ASC after rVSV immunization was not significantly different than that produced by wild type animals, and caspase-1−/− and ASC−/− mice were only partially protected from rVSV-induced pathology. Those data support the idea that some of the IL-1β expressed *in vivo* in response to VSV may be activated by a caspase-1 and ASC-independent mechanism. Together these results suggest that rVSV vectors engineered to suppress the induction of IL-1β, or signaling through the IL-1R would be less reactogenic *in vivo*, but would retain their immunogenicity and protective capacity. Such rVSV would be highly desirable as either vaccine vectors or oncolytic therapies, and would likely be better tolerated in human vaccinees.

## Introduction

Vaccines based on attenuated viral vectors (e.g. poxviruses, adenovirus) are highly immunogenic, relatively inexpensive to produce, and can be delivered by multiple routes. These advantages have accelerated the development of virus-vectored vaccines, especially as concerns about emerging infectious diseases such as avian influenza and multi-drug resistant tuberculosis increase. However, pre-existing anti-vector immunity can significantly impair the ability to raise immune responses to vaccination in the pre-immune host. That suggests that it will be advantageous to prioritize development of viral vaccine vectors to which pre-existing immunity is rare in the human population. Vaccine vectors based on recombinant vesicular stomatitis virus (rVSV) are highly immunogenic and can protect small animals and non-human primates against a range of infectious diseases [1], [2], [3], [4], [5]. VSV is also a potent oncolytic agent, and has been shown to be superior to nine other viruses tested against multifocal glioma [6], [7] and other cancers [8], [9]. Natural infection of humans with VSV is extremely uncommon, and therefore pre-existing immunity to rVSV vectors is almost non-existent [10], [11], [12]. Despite these advantages, development of rVSV for human clinical use continues to be delayed because of concerns about vector-associated pathology. Small laboratory animals immunized with rVSV vaccines display clinical signs of acute illness and lose up to 20% of their pre-immunization body weight in the first four days after immunization [13], [14]. Although most animals recover, undesirable side effects such as these would be unacceptable in human vaccinees. Weight loss is less pronounced in non-human primates immunized with rVSVs, but the one human to date therapeutically immunized with a single cycle non-replicating experimental rVSV vaccine against Ebola virus developed a high fever and transient viremia in the first 24 hours after vector administration [15]. That result demonstrated that even single cycle vectors can induce significant adverse side effects, and suggested that alternate strategies to reduce residual viral vector reactogenicity are required.

For rVSV-based vaccines to move forward to clinical use it is important to identify ways to eliminate rVSV vector-associated pathology that do not at the same time compromise vector immunogenicity or oncolytic properties. It has been shown previously that TNF-α produced in response to intranasal immunization with rVSV vaccine vectors contributes to, but is not the only factor responsible for, rapid weight loss after rVSV immunization [14]. In those studies, TNF-α deficient mice were partially protected from acute pathology after VSV challenge, and TNF-α was not required for the generation of humoral immune responses to rVSV [14]. We undertook the present study to expand on those observations, focusing on the importance of IL-1. We addressed intramuscular immunization, because that is the most likely route of administration for rVSV vectors in humans.

## Results

### Mice deficient in the interleukin-1 receptor (IL-1R−/−) are protected from weight loss after immunization with VSV vaccine vectors

**Figure 1:**
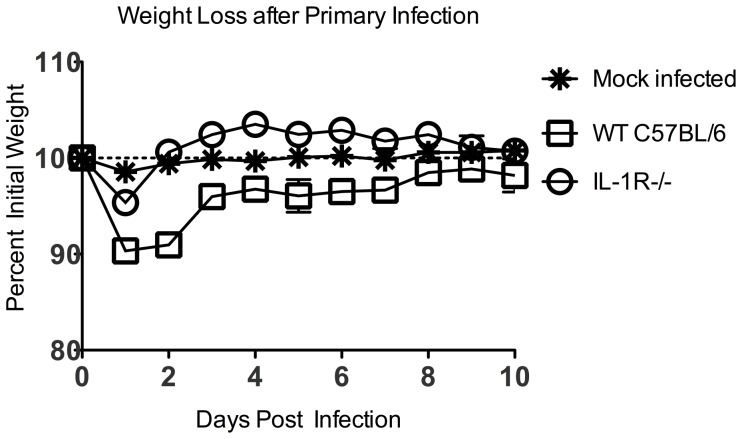
Mice deficient in the interleukin-1 receptor (IL-1R−/−) are protected from acute weight loss after intramuscular immunization with rVSV. Eight to ten week old female C57BL/6 mice were immunized with a single intramuscular injection to the left rear quadriceps of 5×10^8^ PFU of live replicating rVSV. Graph shows average percent initial weight by day for each group of mice beginning on the day of immunization (Day 0). IL-1R−/− mice (n = 3) lost less weight and recovered more quickly than wild type (WT, n = 4) mice after immunization. The difference in weight loss was significant from day 1-day 4 after challenge (P = 0.03, Mann-Whitney test). This experiment has been performed two times with consistent results.

Mice immunized with recombinant VSV (rVSV) vaccine vectors lose weight rapidly after immunization. Weight loss is usually maximal by the second day after immunization but most animals recover, with weight returning to normal by five to seven days after immunization. Interleukin-1β (IL-1β) is a pro-inflammatory cytokine that causes acute weight loss and fever in mice and humans [16], [17], [18], and which stimulates the production of other pro-inflammatory mediators such as IL-6 [17], [19] and prostaglandin E2 (PGE_2_) [20], [21]. To determine whether IL-1 can contribute to the induction of acute pathology after administration of rVSV vaccine vectors, we immunized groups of C57BL/6 wild type (WT, n = 4) or IL-1 receptor deficient (IL-1R−/−, n = 3) mice with a single intramuscular injection of 5×10^8^ PFU live replication-competent rVSV. All animals survived the immunization, but wild type mice lost approximately 10% of their pre-immunization body weight by the first day after immunization (Figure 1) and did not fully regain their pre-immunization weight until eight days after immunization. In contrast, IL-1R−/− mice had minimal pathology, losing only a small amount (<5%) of weight on the first day after immunization and returning to their starting weight by the second day after immunization. IL-1R−/− mice lost significantly less weight than wild type control animals after challenge (P = 0.03 from day 1-day 4 after challenge, Mann-Whitney test). Because mice deficient in the IL-1R cannot respond to IL-1, this result demonstrated that either IL-1α or IL-1β, both of which bind to the IL-1R, contributed to acute pathology after rVSV immunization.

### Mice immunized with VSV vaccine vectors produce IL-1β systemically and at the injection site

**Figure 2:**
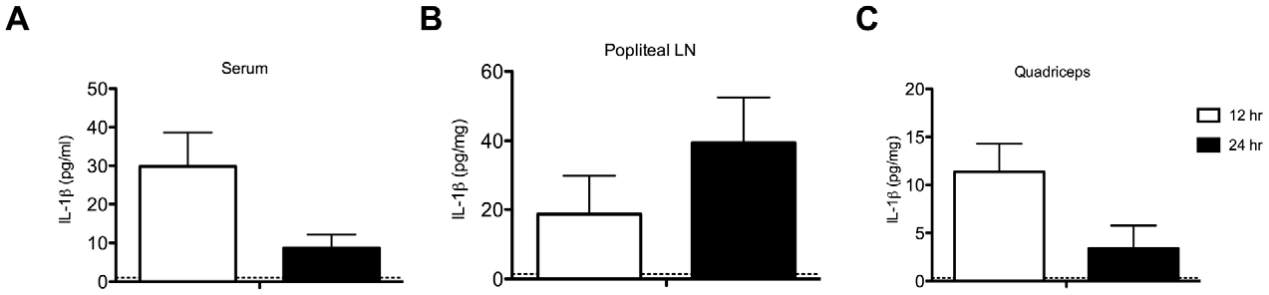
Wild type mice challenged with rVSV produce IL-1β locally and systemically. Groups of adult female C57BL/6 mice were immunized with two injections (5×10^8^ PFU per injection) of rVSV in each rear quadriceps, or were sham inoculated with sterile PBS. At 12 and 24 hours after infection mice (n = 4 per timepoint), were sacrificed and IL-1β in the blood (Panel A), draining popliteal LN (Panel B), and quadriceps muscle (Panel C) was quantitated via ELISA. Open and filled bars represent the 12 and 24 hour timepoints respectively. Dotted line on graphs shows the average amount of IL-1β detected in the blood and tissues of sham-inoculated mice (n = 4). This experiment has been performed twice with consistent results.

Both IL-1α and IL-1β are regarded as pro-inflammatory cytokines, but IL-1β rather than IL-1α has been more commonly associated with symptoms such as weight loss and fever that are induced by infection with a live virus or other immune stimulus [16], [17]. To determine whether intramuscular immunization with VSV vaccine vectors induced production of IL-1β *in vivo* we challenged wild type C57BL/6 mice with rVSV and used an ELISA to quantitate the amount of IL-1β produced locally (at the injection site and in the draining lymph node) and systemically (in the blood). As shown in Figure 2A–C, mice immunized intramuscularly with rVSV had accumulations of IL-1β in the blood, in the quadriceps muscle, and in the popliteal lymph node that drains the quadriceps at 12 and 24 hours after immunization with VSV rwt. These results confirmed that IL-1β was produced *in vivo* both locally and systemically after intramuscular rVSV challenge, and was consistent with the results of Poeck et al. who found that wild-type mice injected intravenously with rVSV had detectable levels of IL-1β in the serum at six hours after challenge [22]. Our results were also consistent with the hypothesis that IL-1β caused the acute pathology that occurs after immunization with rVSV vaccine vectors, and suggested that reducing the production of IL-1β after rVSV immunization might correspondingly reduce reactogenicity and other undesirable side effects of vaccination.

### Mice deficient in the IL-1R control rVSV replication, have normal cellular and humoral immune responses, and are immune to high dose re-challenge

**Figure 3:**
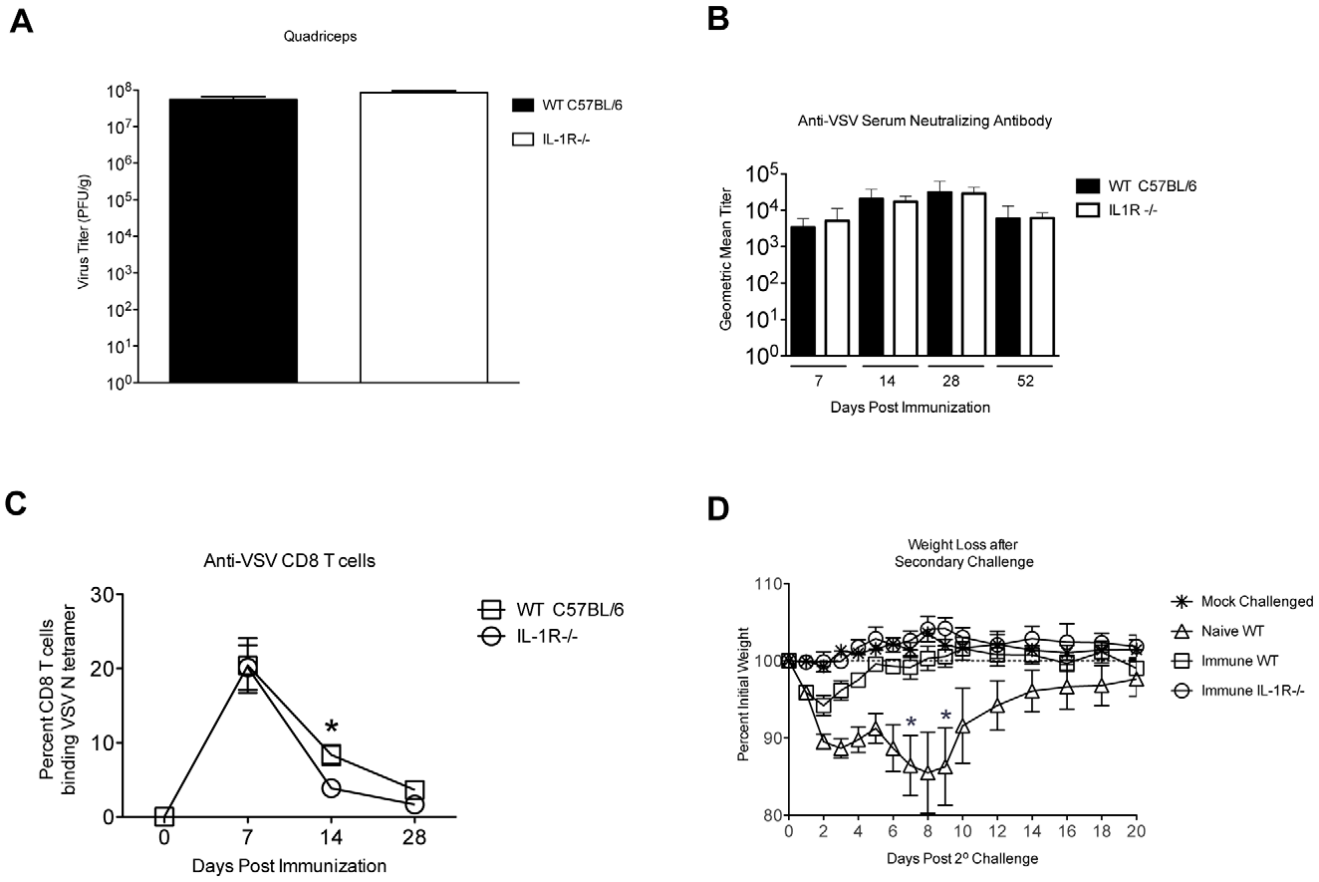
IL-1R−/− mice control VSV replication, make strong humoral and cellular immune responses, and are immune to rechallenge. Adult female wild type or IL-1R−/− (n = 3 per group) mice were immunized intramuscularly with a single injection of 5×10^8^ PFU rVSV in the rear quadriceps. Mice were sacrificed at 24 hours after infection and viral titers determined in the quadriceps muscle (Panel A). There was no significant difference in viral loads between the two groups. In Panels B–D, adult female wild type (n = 5) or IL-1R−/− (n = 4) mice were immunized intramuscularly with a single injection of 5×10^8^ PFU rVSV in the rear quadriceps. At the indicated timepoints after infection mice were bled and humoral and cellular immune responses were assayed. Panel B shows average anti-VSV neutralizing antibody responses by group as measured by microneutralization assay. Error bars represent the upper and lower limits of the 95% confidence interval. Panel C shows average percent CD8 T cells specific for the VSV N1 epitope as measured by MHC Class I tetramer. At 14 days after immunization, WT mice had significantly more (P = 0.03, Two-tailed T test) VSV N specific CD8 T cells than IL-1R−/− mice, but by day 28 the difference was no longer significant (P = 0.13). At eight weeks after the primary infection all mice were challenged intranasally with a semi-lethal dose of rVSV (1×10^8^ PFU). A cohort of naïve wild type mice (n = 7) was challenged at the same time. All pre-immune mice had robust immunity to rechallenge (Panel D) and did not lose weight or exhibit other signs of pathology. Two of the naïve mice succumbed to infection. Days on which naïve animals succumbed are indicated with an asterisk on the graph.

IL-1β causes some deleterious effects such as fever and anorexia, but has also been shown to contribute positively to the generation of adaptive immune responses induced by live virus infection [23]. The role of IL-1 in control of VSV replication has not been examined previously, and reports describing the role of IL-1 in control of other viruses *in vivo* are not in agreement. Because our ultimate goal is to be able to generate less reactogenic rVSV vectors by attenuating the host inflammatory response, it was important to determine whether IL-1 was required for the control of vaccine vector replication, generation of humoral and cellular immune responses, or to generate protection against rechallenge. To investigate these questions we intramuscularly immunized mice deficient in the IL-1 receptor (IL-1R−/−) with 5×10^8^ PFU of rVSV and measured viral loads, induction of antibody and T cell responses, and protection from rechallenge. As shown in Figure 3A, viral loads in the quadriceps muscle (injection site) were not significantly different between wild type and IL-1R−/− mice (n = 3 per group) at 24 hours after infection, which is the peak of VSV replication *in vivo*. Also, virus was not detected in the blood of any infected animal. Those results demonstrated that IL-1 is not required for the control of VSV replication *in vivo*. As shown in Figure 3B, serum neutralizing antibody titers against VSV were not significantly different in IL-1R−/− (n = 4 per group per timepoint) and wild type mice (n = 5 per group per timepoint) at any time after immunization. When we used an MHC Class I tetramer to measure CD8 T cell responses to an immunodominant H-2 K^b^-restricted epitope (N-RGYVYQGL-C) in the VSV N protein [24], IL-1R−/− mice had slightly fewer anti-VSV N specific CD8 T cells in the blood than did wild type animals (Figure 3C) at 14 and 28 days after immunization, although the difference was only statistically significant at 14 days after immunization (P = 0.03, Two-tailed T test). Finally, to determine whether these immune responses were sufficient to protect wild type and IL-1R−/− mice from re-challenge with rVSV, we challenged all animals intranasally with 1×10^8^ PFU of rVSV at eight weeks after the primary infection. As shown in Figure 3D, pre-immune wild type and pre-immune IL-1R−/− mice were fully protected from rechallenge, while naïve wild type animals (n = 7, open triangles, Figure 3D), lost up to 20% of their pre-infection body weight. Two out of the seven naïve animals succumbed to infection. Together, these results indicated that IL-1 was not required either for the control of rVSV replication *in vivo*, or for the generation of protective anti-VSV immune responses. The results also supported the idea that suppressing the production of or response to IL-1β in response to rVSV vector administration would not render rVSV vectors unsafe or non-immunogenic.

### Mice deficient in inflammasome adaptor molecule ASC are partially protected from acute weight loss after immunization with rVSV vaccine vectors

Because the ultimate goal of these studies was to devise strategies by which we could suppress IL-1β production and thereby reduce the pathology of rVSV vectors *in vivo,* we sought to determine the mechanism by which IL-1β was being produced in response to VSV. IL-1β is synthesized as an inactive precursor molecule (pro-IL-1β), which must be cleaved either intracellularly by endogenous protease caspase-1 [25], [26], [27], or extracellularly by matrix metalloprotease 9 [28] or other neutrophil [29] and mast cell-associated proteases [30] to become biologically active. It was shown recently that murine bone marrow derived dendritic cells (BMDC) infected with VSV *in vitro* produce IL-1β via formation of an inflammasome composed of RNA helicase RIG-I, adaptor molecule ASC, and caspase-1 [22]. Because the authors did not test whether RIG-I, ASC, and caspase-1 were required to produce IL-1β in response to VSV *in vivo* we used caspase-1-deficient and ASC-deficient mice to determine the effects of the absence of these molecules on acute pathology after rVSV immunization. It was not practical to test RIG-I deficient mice for IL-1β induction, because RIG-I deficient mice do not produce IFN in response to VSV and therefore rapidly succumb to infection [31].

**Figure 4:**
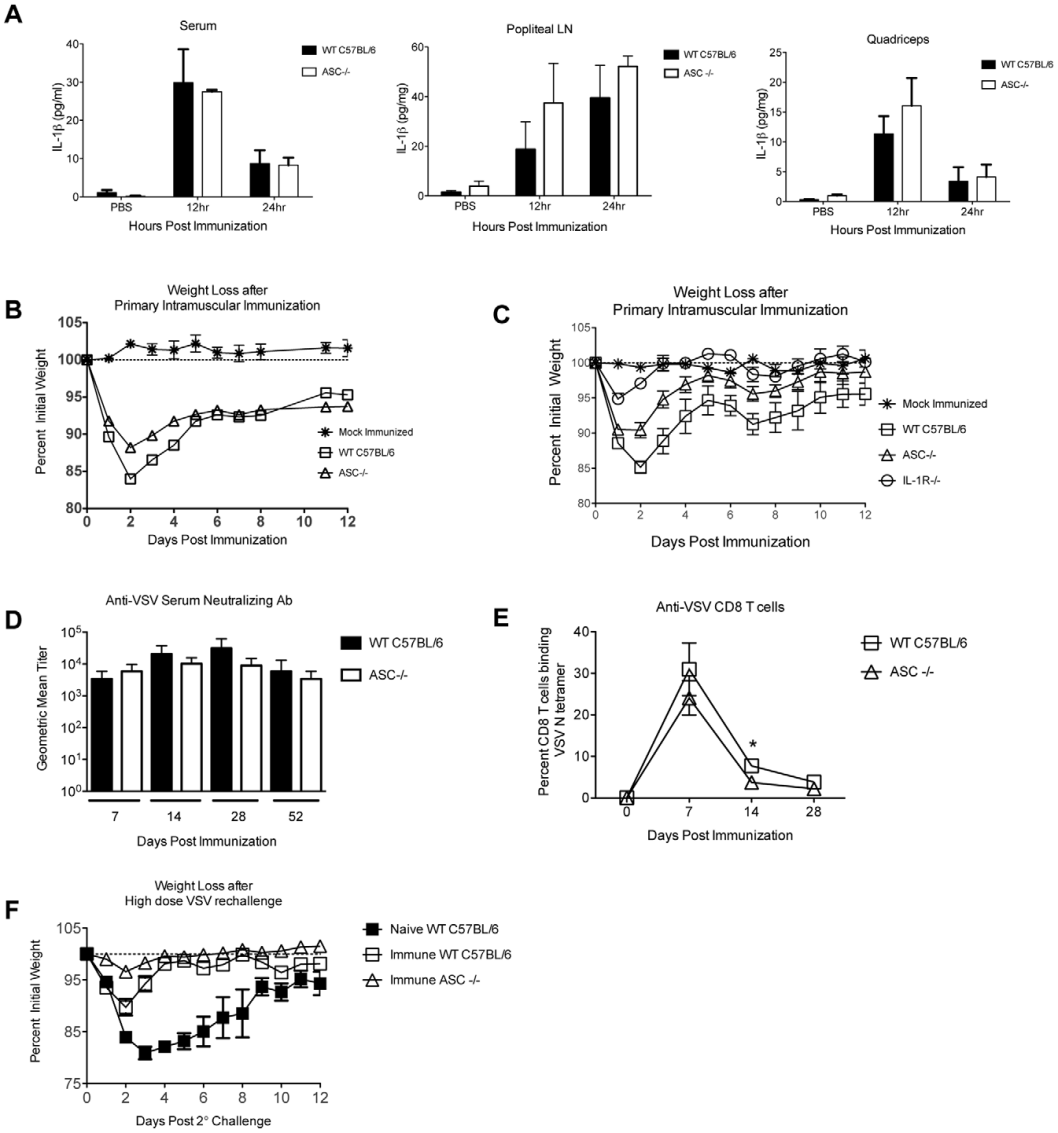
Mice deficient in the inflammasome adaptor ASC (ASC−/−) are partially protected from acute weight loss after intramuscular immunization with rVSV. Groups of adult female C57BL/6 wild type or ASC−/− mice were immunized with two injections (5×10^8^ PFU per injection) of rVSV in each rear quadriceps, or were sham inoculated with sterile PBS. At 12 and 24 hours after infection mice (n = at least 4 per timepoint), were sacrificed and IL-1β in the blood (Panel A left), draining popliteal LN (Panel A middle), and quadriceps muscle (Panel A right) was quantitated via ELISA. The amount of IL-1β produced by wild type and ASC−/− mice was not significantly different at any time or in any organ. The comparison of IL-1β production by wild type and ASC−/− mice has been performed twice with consistent results. Panel B shows average percent initial weight for wild type (n = 6) and ASC−/− (n = 5) mice after intramuscular challenge with 5×10^8^ PFU of rVSV. The comparison of WT and ASC−/− mice has been performed four times with consistent results. Panel C shows average percent initial weight for wild type (n = 5), ASC−/− (n = 5), and IL-1R−/− (n = 4) mice infected with rVSV. IL-1R−/− mice lost significantly less weight than wild type (days 2–4) or ASC−/− mice (days 1–2) (P<0.05 via one way ANOVA with Bonferroni test). The comparison of WT, IL-1R−/−, and ASC−/− mice has been performed twice with consistent results. Panel D shows average serum neutralizing titers for WT (n = ) and ASC−/− (n = ) mice after primary immunization with VSV. There were no significant differences in neutralizing titer between the two groups. Panel E shows average percent CD8 T cells specific for the VSV N1 epitope as measured by MHC Class I tetramer. At 14 days after immunization, WT mice (n = 9) had significantly more (P = 0.001, Two-tailed T test) VSV N specific CD8 T cells than ASC−/− mice (n = 9), but by day 28 the difference was no longer significant (P = 0.07). At eight weeks after the primary infection pre-immune WT (n = 10) and ASC−/− (n = 6) mice were challenged intranasally with a semi-lethal dose of rVSV (1×10^8^ PFU). A cohort of naïve wild type mice (n = 5) was challenged at the same time. All pre-immune mice had robust immunity to rechallenge (Panel F) and did not lose weight or exhibit other signs of pathology. One of the naïve mice succumbed to infection.

We challenged ASC-deficient mice (ASC−/−) and wild type C57BL/6 mice intramuscularly with 5×10^8^ PFU rVSV as in Figures 1, 2, and 3 and measured production of IL-1β and acute pathology after immunization. As shown in Figure 4A, the systemic and local production of IL-1β was not significantly reduced in ASC−/− mice relative wild type control mice immunized in parallel. Consistent with that result, and with our prediction that IL-1β induces acute weight loss after rVSV infection, ASC−/− (n = 5) mice lost slightly less weight after rVSV challenge than did wild type C57BL/6 (n = 6) mice infected in parallel, with the difference only reaching statistical significance only on the second day after challenge (P = 0.004, Mann Whitney Test, Figure 4B). A second experiment replicated these findings almost exactly, with ASC−/− mice (n = 6) showing slightly enhanced protection relative wild type animals (n = 10, Figure S1). The difference in weight loss between wild type and ASC−/− mice was small but highly reproducible, and ASC−/− mice were never protected to the same extent as IL-1R−/− mice. To confirm that observation, we infected the three groups in parallel (5×10^8^ PFU rVSV intramuscular). As shown in Figure 4C, IL-1R−/− mice (n = 4) lost significantly less weight than wild type (n = 5) mice (days 1–4 after challenge P<0.05 via one way ANOVA with Bonferroni multiple comparison test) and recovered their pre-immunization body weight more quickly than either wild type or ASC−/− mice. The difference in weight loss between IL-1R−/− and ASC−/− mice was significant on the first and second day after challenge (P<0.05 via one way ANOVA with Bonferroni multiple comparison test). Similar to the results obtained in IL-1R−/− mice, ASC−/− mice made equivalent humoral responses and slightly reduced cellular responses to VSV, and were fully protected from high dose rechallenge (Figure 4D–F). Taken together, these results demonstrated that production of IL-1β *in vivo* after intramuscular rVSV immunization occurred independent of inflammasome adaptor molecule ASC. Because ASC deficient mice were not protected fully from acute pathology after rVSV immunization, strategies that suppress the function of ASC would be predicted to partially but not completely abrogate acute pathology after rVSV immunization.

### Mice deficient in caspase-1 are partially protected from acute weight loss after immunization with rVSV vaccine vectors

**Figure 5:**
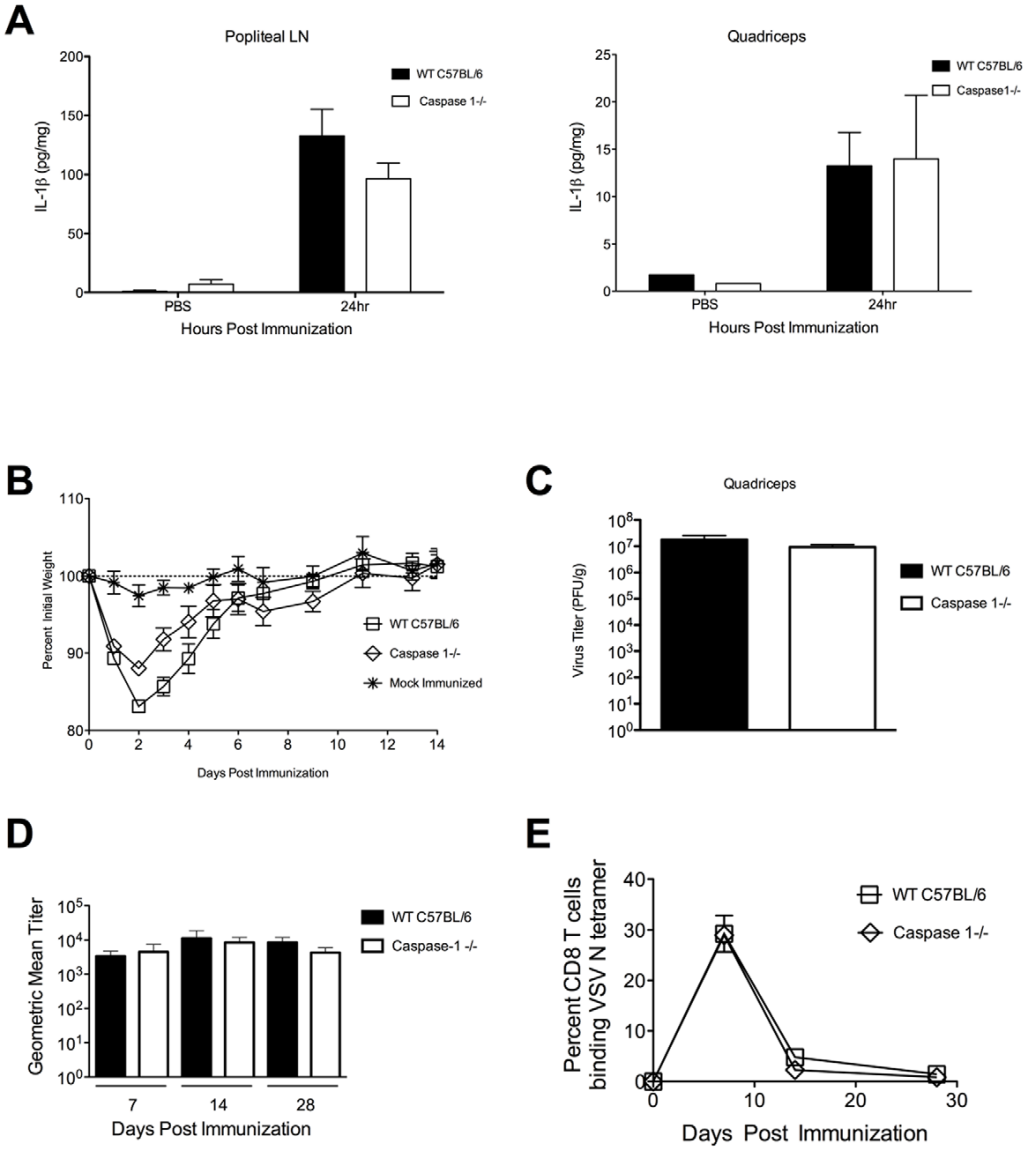
Mice deficient in caspase-1 are partially protected from acute weight loss after intramuscular immunization. Groups of adult C57BL/6 wild type or caspase 1−/− mice were immunized with two injections (5×10^8^ PFU per injection) of rVSV in each rear quadriceps, or were sham inoculated with sterile PBS. At 24 hours after infection mice (n = 2–3 mice per timepoint), were sacrificed and IL-1β in the draining popliteal LN (Panel A left) and quadriceps muscle (Panel A right) was quantitated via ELISA. The amount of IL-1β produced by wild type and caspase 1−/− mice was not significantly different in either organ. The comparison of IL-1β production by wild type and caspase 1−/− mice has been performed twice with consistent results. Panel B shows average percent initial weight for wild type and caspase 1−/− mice (n = 5 per group) after intramuscular challenge with 5×10^8^ PFU of rVSV. The comparison of WT and caspase 1−/− mice has been performed twice with consistent results. Caspase 1−/− mice lost significantly less weight than wild type controls on days 2 and 3 after challenge (P<0.05 via Mann Whitney test). Panel C shows average viral loads in the quadriceps muscle of infected mice (n = 6 per group). Data is compiled from two identically performed experiments. Viral loads in WT and caspase 1−/− mice were not significantly different. Panel D shows average serum neutralizing antibody titers for wild type and caspase 1−/− mice immunized with rVSV (n = 5 per group per timepoint). Error bars represent the upper and lower limits of the 95% confidence interval. Panel E shows average percent ± SEM of CD8 T cells in the blood binding to an MHC Class I tetramer recognizing an immunodominant epitope within VSV N. There were no significant differences in the antibody or CD8 T cell responses between the two groups at any time. The comparison of humoral and cellular immune responses in WT and caspase 1−/− mice has been performed twice with consistent results.

Similarly, when we immunized mice deficient in caspase-1 (caspase-1−/−) or wild type mice with 1×10^9^ PFU of rVSV, caspase-1 deficient mice did not have significantly reduced levels of IL-1β relative wild type mice (Figure 5A) when we measured IL-1β production at the injection site and in the draining lymph node by ELISA. Consistent with those data, caspase-1 deficient mice immunized intramuscularly with 5×10^8^ PFU of rVSV as in Figure 4B–C (n = 5 per group), were partially but not completely protected from acute weight loss (Figure 5B) relative wild type mice (n = 5), with the difference in weight loss between wild type and caspase-1−/− mice being significant only on days 2 and 3 after challenge (P<0.05, Mann Whitney test). Caspase-1 deficient mice controlled viral replication as well as wild type mice (Figure 5C, n = 6 per group), with no significant difference in viral loads in the quadriceps muscle of infected animals at 24 hours after infection. As with IL-1R−/− mice, no virus was recovered from the blood of infected mice. Caspase-1 deficient mice made robust humoral (Figure 5D) and cellular (Figure 5E) responses to VSV, which were not significantly different than those of wild type mice.

## Discussion

We undertook this study with the goal of determining which cytokines induced by rVSV contribute to acute pathology after intramuscular immunization. This is important because while VSV is a highly promising vaccine vector and oncolytic agent, its clinical development has lagged behind that of other live viruses because of concerns about vector-associated pathology. Fever, myalgia, and the “sickness response” are induced by many live viral or bacterial vaccines [32], [33], [34], [35], [36], and these vaccine-induced side effects are among the leading reasons why some individuals elect not to receive protective vaccines [37], [38], [39]. Consistent with our results obtained in the mouse model and presented here, several recent studies have correlated increased levels of IL-1 or other pro-inflammatory cytokines with the induction of high fevers or other adverse events in response to live virus vaccination in humans [36], [40], or have identified genetic polymorphisms in the IL-1 gene which predispose individuals to severe adverse events after receiving live virus vaccines [41]. For these reasons it is important to determine the ways in which pro-inflammatory cytokines contribute to reactogenicity of VSV vectors in particular, not only because those findings could advance development of VSV-based therapeutics, but also because our findings might help to inform the development of other live virus vaccines and oncotherapies.

Previous studies in which VSV vectors were delivered to mice intranasally showed that intranasal immunization with VSV induces the pro-inflammatory cytokine TNF-α, and that TNF-α production directly correlated with weight loss and acute pathology [14]. Although intranasal immunization has many advantages (needle free delivery, induction of mucosal immunity, etc), a significant drawback to intranasal immunization with rVSV is the risk of neurotropic spread of the virus. VSV instilled in the nose rapidly colonizes the olfactory neurons, and migrates into the brain [42]. The neurovirulence of VSV vectors has been significantly reduced by attenuating the capacity of the VSV vector to replicate [43], but even a remote chance of neurotropic spread will likely prevent use of the intranasal route for human inoculations. Therefore, we decided to determine which cytokines were responsible for acute pathology after intramuscular immunization, which is regarded as a safer route by which to administer potentially neurotropic agents.

We show here that mice deficient in the interleukin-1 receptor (IL-1R) are significantly protected from weight loss after intramuscular challenge with VSV. Although these results do not preclude the contribution of other inflammatory processes to pathology, they do positively identify IL-1 as an important target for intervention.

The IL-1 receptor binds IL-1α and IL-1β. Therefore because IL-1R−/− mice were protected from pathology, it was possible that IL-1α, IL-1β, or both cytokines contributed to VSV-associated pathology *in vivo*. IL-1β has been well characterized as a mediator of acute inflammatory responses in mice and humans, namely the induction of fever and cachexia, the acute phase response, and upregulation of other inflammatory mediators such as IL-6 in response to infectious or non-infectious stimuli [44], [45], reviewed in [46]. IL-1β is more commonly associated with these pathologies than is IL-1α. For example, IL-1β deficient mice, but not IL-1α deficient mice, are protected from fever after injection of turpentine [16], [17]. Similarly, IL-1β, but not IL-1α, is the etiologic agent of hereditary periodic fever syndromes and other “autoinflammatory” diseases in humans [47], [48]. IL-1α is more commonly associated with “sterile” inflammation that accompanies apoptotic cell death [49], and with the exception of adenovirus mediated inflammation [50] has not been associated with pathology arising from viral infection. For these reasons, we predict that IL-1β is the primary mediator of pathology in our system, but further experiments will be required to formally exclude a role for IL-1α.

Once we had established that IL-1 contributed to acute pathology after intramuscular VSV inoculation, the most important question to pursue was whether IL-1 would be required for control of VSV replication *in vivo*, and for the induction of immune responses to the VSV vector. If IL-1 were not required for control of virus replication, or for generation of protective immune responses, then that would suggest that engineering rVSV vectors to suppress IL-1 production and/or signaling would be a rational approach to reducing rVSV associated pathology.

Although three separate reports have now described the induction of IL-1 in response to VSV challenge *in vivo* [22], [51], [52], none of these had examined whether or not the induced IL-1 contributed positively to or was required for generation of immune responses or protection. In other viral infection models where this has been investigated, published reports are not in agreement. For example, Schmitz et al found that IL-1R−/− mice infected with influenza had a significantly higher rate of mortality than did wild type control animals, but the increase in mortality did not correlate with a higher virus burden in the IL-1R−/− mice [53]. Three additional reports examined the role of IL-1β, as well as components of the Nlrp3 inflammasome, in control of influenza infection [23], [54], [55]. In these, two out of three found transiently elevated viral loads in mice in which IL-1β production was decreased [23], [54], the other report found no difference in virus burden [55]. Similarly, only one of the three reports [23] found that a reduction in IL-1β correlated with a reduction in cellular immune responses- the others either did not examine immune responses or did not find a correlation.

In this study, we found that IL-1R−/− mice controlled viral loads as well as wild type control animals. Viral loads at the injection site were not significantly different between groups, and neither group had a detectable viremia after challenge. This result highlights the relative safety of the intramuscular immunization route. Equally important was that IL-1R−/− mice challenged with VSV made robust humoral and cellular responses to VSV antigens, and were fully protected from a high dose intranasal rechallenge with VSV. Interestingly, we did observe levels of anti-VSV CD8 T cells in the IL-1R−/− mice and in ASC−/− mice were transiently but significantly decreased, which is consistent with the report of Iwasaki et al, who found that anti-influenza CD8 T cell responses were slightly decreased in mice with reduced levels of IL-1β production [23]. In our studies, the number of anti-VSV specific CD8 T cells was significantly lower in IL-1R−/− and ASC−/− mice at 14 days after infection, but was not significantly different at either 7 or 28 days after infection. This finding warrants further investigation, and follow-up studies will focus on determining whether CD8 T cells primed in the absence of IL-1 signaling are functionally different (e.g. in cytotoxic capacity, or in acquisition of central memory phenotype) from those primed in IL-1 intact animals.

In summary, these results support the idea that IL-1 production is not required either for control of VSV replication *in vivo*, or for the induction of protective immune responses to VSV antigens after intramuscular immunization. Because the relative magnitude of immune response to foreign antigens expressed by VSV is generally similar to the relative magnitude of the immune response to VSVs own antigens [56], we predict that IL-1 would also not be required for the induction of immune responses to vaccine antigens expressed by VSV. Nonetheless, determining whether IL-1 is important for the response to foreign (vaccine) antigens expressed by an rVSV will be an important direction for further study.

The second question arising from our results is how the VSV-induced production of IL-1 might be suppressed. One means of suppressing the biological response to IL-1 in humans is via injection of recombinant IL-1 receptor antagonists. Currently marketed as the drugs Anakinra or Kineret, these agents effect a systemic suppression of IL-1α and IL-1β. Although systemic suppression of IL-1 is an effective treatment for some conditions (severe rheumatoid arthritis, for example) [57], systemic suppression of IL-1 also carries the risk of enhanced susceptibility to infection [57], [58]. Therefore we decided to determine whether there might be a way of suppressing IL-1 only in the cells directly infected with VSV, or within the focus of VSV-infected cells. To determine the appropriate target molecule(s) to effect local suppression of IL-1, it was necessary to determine the mechanism by which IL-1 was being produced in our model. Several years ago, Muruve et al reported that adenovirus activated human monocytes (THP-1 cells) to produce IL-1β *in vitro*, and that IL-1β production was dependent upon NLRP3 and caspase-1 [51]. In the supplement to that manuscript, the authors reported that infection of THP-1 cells with VSV did not induce IL-1β production, although detailed methods for that study were not provided. In contrast to that, two more recent studies reported that VSV-infected THP-1 cells do produce IL-1β *in vitro*. In the first study, Poeck et al found that VSV induced production of IL-1β was dependent upon caspase-1, but not upon NLRP3 [22]. In contrast to that, Rajan et al found VSV mediated induction of IL-1β from THP-1 cells to be dependent on NLRP3, and dependent on caspase-1 [52]. It is likely that the discrepancies in these reports were due to subtle differences in experimental procedures, but the different outcomes reported by each of these groups highlight the complexity of elucidating the precise mechanism(s) by which VSV may induce IL-1.

In our studies we found that VSV induced robust production of IL-1β *in vivo* and that IL-1β was still produced in mice lacking caspase-1 or ASC. One caveat to those findings is that ELISA assays such as the one used here do not rigorously discriminate between detection of pro-IL-1β and detection of the cleaved active IL-1β. Therefore it is possible that the some of the IL-1β detected in caspase−/− mice was not biologically active. Precise determination of the amount of active IL-1β present in caspase−/− mice will require the development of better detection reagents. Despite this limitation, our results support a model in which there are multiple pathways by which mature IL-1β is produced *in vivo* in response to VSV. The data are also consistent with the idea that the manner in which mature IL-1β is produced (via caspase-1 or not) may vary with the cell in which the IL-1β is induced. We further observed that mice lacking caspase-1 or ASC were not protected from acute pathology to the same extent that IL-1R−/− mice were. That meant that VSV vectors engineered to suppress components of the RIG-I/ASC/caspase-1 inflammasome, or the Nlrp3/ASC/caspase-1 inflammasome, would be unlikely to be significantly less reactogenic than the parent vector. On the other hand, rVSV designed to suppress the biological response to IL-1β (for example via inclusion of a soluble IL-1β trap), or to IL-1α and IL-1β (via inclusion of a soluble IL-1 receptor antagonist) might reduce the biological response to IL-1 *in vivo* and therefore be less reactogenic.

In summary, we have shown here that IL-1 contributes to acute pathology after intramuscular immunization with VSV. IL-1 was not required for control of viral replication, for the induction of cellular or humoral immune responses, or for development of protective immunity to rechallenge. These results add to our understanding of the role for IL-1 in promoting immunity to viral challenge, and support the notion that the requirement for IL-1β in promoting adaptive immunity may vary according to the type and dose of pathogen encountered as well as the route of exposure. Finally, by identifying IL-1β as a major source of reactogenicity for rVSV vaccines, we are able to propose a novel strategy to ameliorate side effects without compromising immunogenicity.

## Materials and Methods

### Ethics Statement

All animal studies were reviewed and approved by the Duke University Institutional Animal Care and Use Committee.

### Viruses

Vesicular stomatitis virus (Indiana strain) was originally obtained from Dr. John Rose (Yale University). Virus was propagated on BHK-21 cells (ATCC CCL-10) and titered using a standard plaque assay.

### Inoculation of mice

Eight to ten-week-old C57BL/6 wildtype and IL-1 receptor type 1 deficient (IL-1R−/−, strain name B6.129S7-*Il1r1^tm1Imx/J^*) mice were obtained from Jackson Laboratories. Caspase1−/− on the C57BL/6 background were generously provided by Dr. Fayyaz Sutterwala and Dr. Richard Flavell. ASC−/− and Nlrp3−/− mice on the C57BL/6 background were generously provided from Genentech Inc. (San Francisco, CA). Mice obtained commercially were housed for at least 1 week before experiments were initiated. Mice were housed in microisolator cages in a biosafety level 2-equipped animal facility. Viral stocks were diluted to appropriate titers in serum-free DMEM. For intramuscular immunization (i.m.), mice were injected with the indicated amount of virus(es) in 50 µl total volume. For intranasal (i.n.) vaccination, mice were lightly anesthetized with isoflurane using a vaporizer and administered the indicated amount of virus in 30 µl total volume. The Institutional Animal Care and Use Committee of Duke University approved all animal experiments.

### Determination of viral titers by plaque assay

Mice were sacrificed via anesthetic overdose and organs removed aseptically. After dissection organs were weighed, and homogenized in sterile buffer (100 µl buffer per 0.1 g organ weight). Homogenates were titered by standard plaque assay on BHK-21 cells (ATCC CCL-10) using a semi-solid overlay to detect infectious VSV. After 48 hours the overlay was removed and the cell layer stained with crystal violet to visualize plaques.

### IL-1β ELISA

Mice were bled and then sacrificed via anesthetic overdose and organs removed aseptically. Organs were homogenized in 500 µL (100 µL for lymph nodes) of buffer containing 137 mM NaCl, 20 mM Tris-Cl pH 8.0, 5 mM EDTA, 0.05% Triton-X 100, and protease inhibitor cocktail (Roche). Serum and organ homogenate supernatants were assayed for IL-1β by ELISA (R&D Systems). Organ IL-1β amounts were normalized to the amount of protein in the samples, as determined using a bicinchoninic acid (BCA) protein assay (Thermo Scientific).

### Assay for neutralizing antibody against VSV

Blood was obtained from mice on days 7, 14, and 28 after vaccination via cheek bleed. Heat inactivated serum was diluted in serum-free DMEM such that the final dilution in the first well of a 96-well plate was 1∶10 for day 7 samples and 1∶100 for day 14 and 28 samples, with subsequent two-fold dilutions. Samples were assayed in duplicate. 100 PFU of rVSV diluted in serum-free DMEM was added to each well and incubated for one hour at 37°C-5%CO_2_, after which 4,000 BHK-21 cells (ATCC CCL-10) diluted in 5%FBS-DMEM were added to each well. Plates were incubated at 37°C-5%CO_2_ for three days, and cytopathic effect was observed. The neutralizing titer was defined as the highest dilution of serum that gave 100% neutralization of rVSV.

### Tetramer assay

To obtain peripheral blood lymphocytes blood was collected into serum free medium (DMEM) containing heparin. Blood was layered onto a Ficoll gradient and spun, after which lymphocytes were collected from the interface. Cells were washed and resuspended in DMEM containing 5% FCS.

Staining was performed on freshly isolated lymphocytes as previously described [4]. Briefly, approximately 5×10^6^ cells were added to the wells of a 96-well V-bottom plate and were blocked with unconjugated streptavidin (Molecular Probes) and F_c_ block (Pharmingen) for 15 min at room temperature (RT). Following a 5-min centrifugation at 500×*g*, lymphocytes were labeled with a FITC-conjugated anti-CD62L antibody, (Pharmingen), an allophycocyanin-conjugated anti-CD8 antibody (Pharmingen), and tetramer for 30 min at RT. The tetramer was a PE-conjugated major histocompatibility complex (MHC) class I K^b^ tetramer (NIH Tetramer Facility) containing the H-2K^b^ restricted peptide VSV N_53–59_ (N-RGYVYQGL-C). Sham-inoculated control animals were used to determine background levels of tetramer binding. Background was routinely less than 0.1% and was subtracted from all reported percentages.

### Statistical Analysis

Statistical comparisons were made using GraphPad Prism software. Results were considered significant when P<0.05.

## Supporting Information

Figure S1 **Mice deficient in the inflammasome adaptor ASC (ASC−/−) are partially protected from acute weight loss after intramuscular immunization with rVSV.** Average percent initial weight for wild type (n = 10) and ASC−/− (n = 6) mice after intramuscular challenge with 5×10^8^ PFU of rVSV. The difference in weight loss between wild type and ASC−/− mice was significant on the first and second day after challenge (P<0.05, Mann Whitney test).
(TIF)
