# Supplementary figures and images for: Acute Reactogenicity after Intramuscular Immunization with Recombinant Vesicular Stomatitis Virus Is Linked to Production of IL-1β
Source: PLoS One. 2012 Oct 8;7(10):e46516. doi: 10.1371/journal.pone.0046516 (PMC3466325; doi:10.1371/journal.pone.0046516)

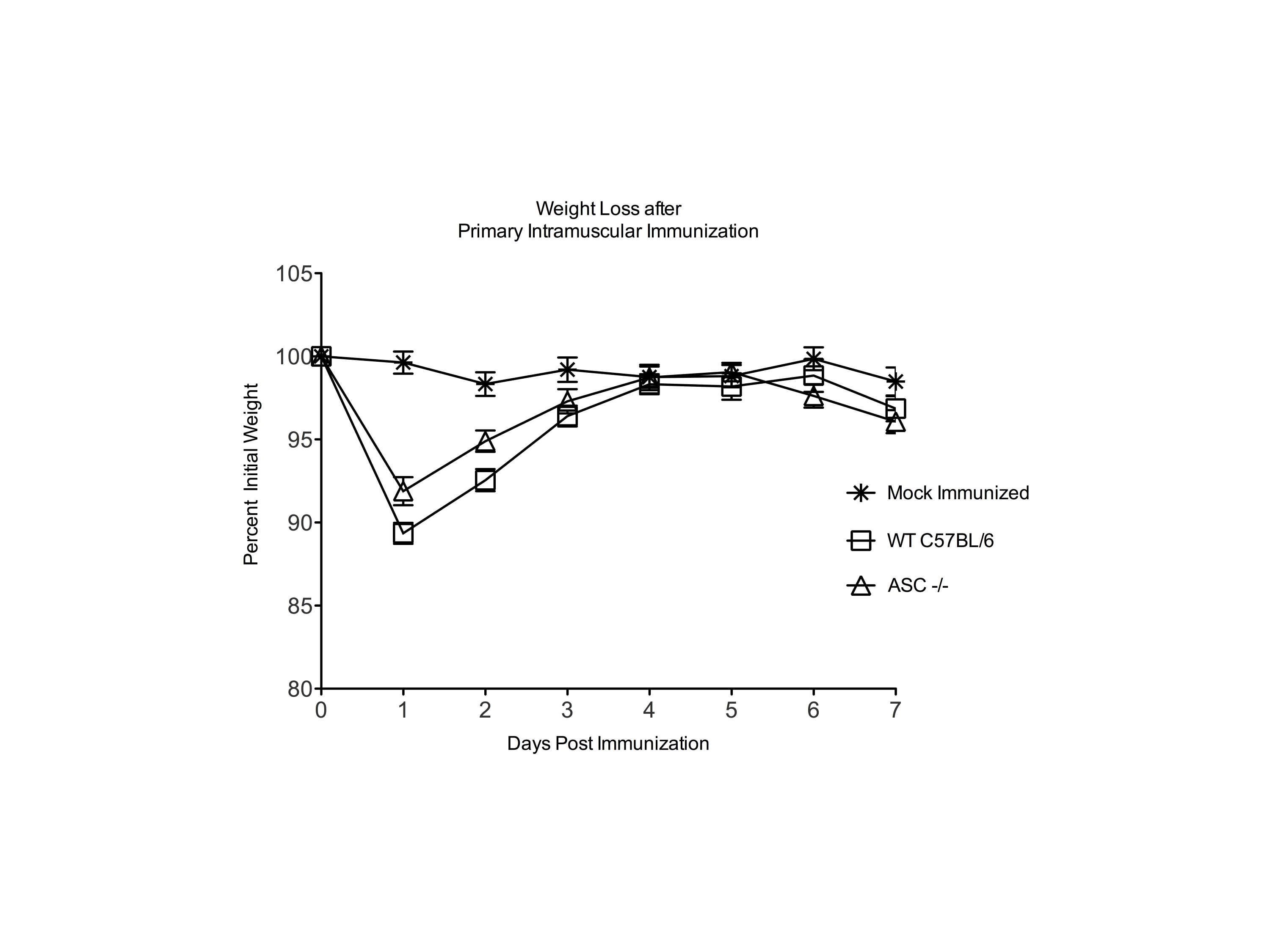

Supplement: Figure S1 — Mice deficient in the inflammasome adaptor ASC (ASC−/−) are partially protected from acute weight loss after intramuscular immunization with rVSV. Average percent initial weight for wild type (n = 10) and ASC−/− (n = 6) mice after intramuscular challenge with 5×10^8^ PFU of rVSV. The difference in weight loss between wild type and ASC−/− mice was significant on the first and second day after challenge (P<0.05, Mann Whitney test). (TIF) [file pone.0046516.s001.tif]
